# Beractant and poractant alfa in premature neonates with respiratory distress syndrome: a systematic review of real-world evidence studies and randomized controlled trials

**DOI:** 10.1038/s41372-020-0603-7

**Published:** 2020-02-12

**Authors:** Manuel Sánchez Luna, Peter Bacher, Kristina Unnebrink, Marisol Martinez-Tristani, Cristina Ramos Navarro

**Affiliations:** 1 Division of Neonatology, Instituto de Investigación Sanitaria Gregorio Marañón, Complutense University of Madrid, Hospital General Universitario “Gregorio Marañón”, O’Donnell 48, 28009 Madrid, Spain; 2 Global Medical Affairs, Pharmaceutical Research and Development, AbbVie Inc., 1 North Waukegan Road, North Chicago, IL 60064 USA; 3 Data and Statistical Sciences, AbbVie Deutschland GmbH & Co. KG, Knollstraße, 67061 Ludwigshafen, Germany

**Keywords:** Therapeutics, Respiratory tract diseases

## Abstract

Findings from previous meta-analyses of randomized clinical trials (RCTs) in premature infants with respiratory distress syndrome (RDS) varied as to whether clinical outcomes differed by type of animal-derived pulmonary surfactant; real-world evidence (RWE) was excluded. We extracted study characteristics and outcomes from full-text articles from a systematic search for studies that compared beractant with poractant alfa for RDS in preterm infants. RWE data were tabulated; RCT data were subjected to meta-analyses. Designs, patient characteristics, and follow-up durations varied widely among studies (4 RWE, 15 RCT). RWE studies with adjusted odds ratios (ORs) found no statistically significant between-treatment differences in outcomes. In RCT meta-analyses, no statistically significant between-treatment differences were observed for death (OR [95% confidence interval], 1.35 [0.98–1.86]), bronchopulmonary dysplasia (1.25 [0.96–1.62]), pneumothorax (1.21 [0.72–2.05]), and air leak syndrome (2.28 [0.82–6.39]). Collectively, outcomes were similar with beractant and poractant alfa in RWE studies and pooled RCTs.

## Introduction

Respiratory distress syndrome (RDS) is common in premature neonates, occurring in ~80% of those born at or <28 weeks gestational age (wGA) [1], and is usually caused by inadequate levels of endogenous lung surfactant [2]. When supportive measures, such as continuous positive airway pressure, are insufficient to maintain oxygenation in preterm infants with RDS, the introduction of exogenous surfactant into the trachea as rescue therapy is an important means of reducing the risk of serious complications and adverse outcomes, such as death, bronchopulmonary dysplasia (BPD), and air leak syndrome (ALS) [1, 3]. Guidelines state that natural (animal-derived) surfactants are preferred over synthetic surfactants because the former are associated with lower rates of adverse outcomes [1, 3].

The two most widely used animal-derived surfactants are beractant and poractant alfa [4]. Beractant is an extract from bovine lungs and contains 25 mg/mL phospholipids, whereas poractant alfa is an extract from porcine lungs and contains 74–76 mg/mL phospholipids; both preparations contain smaller concentrations of natural surfactant-associated proteins and are suspended in 0.9% saline solution [5–8]. Beractant and poractant alfa as rescue therapy for RDS in preterm infants were compared in previous meta-analyses [9, 10]. One meta-analysis found significantly greater rates of death before hospital discharge and of several other endpoints (composite outcome of death or BPD, defined as need for oxygen at 36 weeks postmenstrual age [PMA]; multiple surfactant dosing; and patent ductus arteriosus requiring treatment with a cyclo-oxygenase inhibitor) with beractant compared with poractant alfa; however, no significant difference was found between the two treatments for death before 28 days of age or any other endpoint (BPD at 28–30 days of age, BPD at 36 weeks PMA, pneumothorax, ALS, pulmonary hemorrhage, confirmed bacterial sepsis, necrotizing enterocolitis, periventricular leukomalacia, retinopathy of prematurity, retinopathy of prematurity stage ≥3, intraventricular hemorrhage, and intraventricular hemorrhage stage ≥3) [9]. The other meta-analysis, which focused on mortality, found no significant difference between beractant and poractant alfa for the rate of death [10]. However, neither of these previous meta-analyses aimed to describe real-world evidence (RWE) studies, which might provide valuable additional information on the actual implementation of therapies [11] and are of interest to regulatory authorities [12].

The objective of this analysis was to systematically compare up-to-date published results from peer-reviewed journal articles, both from RWE studies and RCTs, of beractant versus poractant alfa for the rescue treatment of RDS in premature neonates.

## Material and methods

### Literature search

The systematic literature search included the following search terms: (beractant or Survanta) and (poractant or Curosurf) and (RDS) and (comparative study or “vs.” or “versus” or compar* or “head to head”) and (prematur* or preterm or infant or newborn or baby or babies or neonate or birth or childbirth or preemie or <37 weeks [comprising <29, <30, <31, <32, <33, <34, <35, <36, and <37 weeks]). The BIOSIS Previews^®^, Current Contents^®^ Search, Derwent Drug File, Embase^®^, EMCare^®^, International Pharmaceutical Abstracts, MEDLINE^®^, and SciSearch^®^ databases were searched on June 28, 2018; an updated search was conducted on March 27, 2019, but found no additional studies. No limits were applied for publication dates or language.

### Literature selection

Based on abstracts from the literature search, articles about prospective or retrospective clinical studies that compared beractant and poractant alfa for the treatment (but not prevention) of RDS in premature infants were selected. Duplicates, meeting abstracts, review articles, prior meta-analyses, and other inapplicable sources were removed. Full-text versions of the remaining articles were reviewed and those not meeting the study inclusion criteria or not including the endpoints of interest were discarded.

### Data extraction

Data were extracted only from the remaining full-text articles, not from abstracts. When available, the following information was extracted from each article: study design characteristics (e.g., use of blinding, duration of follow-up), key patient eligibility criteria (e.g., gestational age, place of birth, birth weight limit, age at randomization or first dose, level of respiratory support and oxygen parameters, exclusion because of comorbidities), the number of patients in each dose group, patient characteristics (gestational age, weight, cesarean delivery, sex, exposure to prenatal steroids, fractional inspired oxygen [FiO_2_], and the number of times that surfactant was actually administered), definitions of extracted endpoints, and the incidence of endpoints of interest (death, BPD, pneumothorax, and ALS). Study quality was assessed using published grading schemes [9, 13, 14]. The composite endpoint of BPD or death was extracted only for the RWE studies. For the RCTs, the composite endpoint was not extracted because few RCTs reported such data. For RWE studies only, the published odds ratios (ORs), confidence intervals (CIs), and *P* values were extracted to be presented in the current article. Due to the heterogeneity in study designs and adjusted analyses, no meta-analysis was performed on published RWE studies. Results from RWE studies are reported and discussed separately in the current article. If there was ambiguity during data extraction, the authors of the articles were contacted for guidance with proper understanding of their studies.

### Meta-analyses

The primary meta-analyses of RCTs were conducted with all dosing regimens for beractant or poractant alfa aggregated. For trials with multiple dosing arms per product, the respective arms were pooled. A sensitivity analysis included only RCTs or individual RCT treatment arms in which the dosage was consistent with the US product label. The product label for beractant recommends a dose of 4 mL/kg of patient body weight, as often as every 6 h; because the concentration of the surfactant suspension is 25 mg/mL, this corresponds to 100 mg/kg surfactant [5, 7]. The US product label for poractant alfa recommends an initial dose of 2.5 mL/kg, followed by 1.25 mL/kg, which may be administered at ~12-h intervals; because the concentration of the surfactant suspension is 80 mg/mL, this corresponds to 200 mg/kg followed by 100 mg/kg surfactant [6]. Another sensitivity analysis compared equal weight-based doses of beractant and poractant alfa (100 mg/kg each). Finally, sensitivity analyses were conducted to compare the incidence of BPD defined as the need for oxygen at 28 days after birth or as assessed at 36 weeks postconceptional age (PCA) or PMA. The meta-analyses were conducted with a random-effects model using the Mantel–Haenszel method. A random-effects approach was used because of the inherent heterogeneity between the RCTs driven, for example, by different study durations. For the RCTs, the meta-analyses provided ORs, 95% CIs, and *P* values. In some particular cases, risk differences with 95% CIs and *P* values are also reported. All meta-analyses were conducted using Review Manager version 5.3 (Copenhagen: The Nordic Cochrane Centre, The Cochrane Collaboration, 2014).

## Results

### Literature selection

**Fig. 1:**
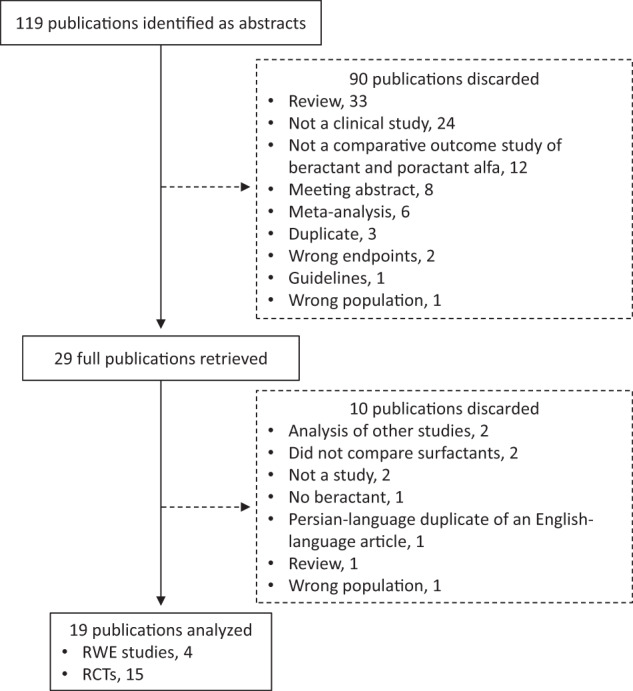
Literature search results. RCT randomized controlled trial, RWE real-world evidence.

The systematic literature search yielded 119 abstracts, of which 90 were discarded (Fig. 1). Twenty-nine full-text articles were retrieved, of which 10 were eliminated. The remaining 19 articles (4 RWE studies [15–18] and 15 RCTs [19–33]) were included.

### RWE studies

#### Study and patient characteristics

Four RWE studies were analyzed (Table 1) [15–18]. Two studies had mid- to long-term outcomes (>72 h) [15, 17], and two studies did not define specific minimum times for patient follow-up [16, 18]. The criterion for gestational age was <37 wGA in two studies [15, 18] <32 wGA in 1 study [17], and 25–32 wGA in another study [16]. Only 1 study had a limit (500–1999 g) for birth weight [16]. The dosages were according to the US product label (i.e., beractant 100 mg/kg; poractant alfa 200 mg/kg for the first dose; and 100 mg/kg for subsequent doses) in 1 study [17], beractant 100 mg/kg and poractant alfa 100 mg/kg for all doses in another study [18], and unspecified in two studies [15, 16]. Although not explicitly stated, it is expected that in the RWE studies, approved doses were used. Two studies included large numbers of patients (51,282 and 14,173) [15, 16], whereas the other two studies were of more modest sizes (415 and 242) [17, 18]. Median or mean gestational age ranged from ~27 to 30 weeks. Median or mean birth weight ranged from 1037 to 2026 g among studies, trending higher with greater gestational age. The proportions of patients who were delivered by cesarean section ranged from 56.1% to 82.6%. The proportion of male patients ranged from 51.6% to 64.0%. The use of prenatal steroids ranged from high to nearly universal (range, 63.7–95.9%). Baseline values for FiO_2_ were not reported. One study reported data on repeat dosing, which was comparable between the two treatment groups (mean number of doses: beractant, 1.78; poractant alfa, 1.63) [17]. Quality assessment of RWE studies is presented in Supplementary Table 1.

**Table 1:** Real-world evidence studies.

Study	Follow-up	Key criteria	Population	Characteristics	Outcomes				
					Death	BPD or Death	BPD	Pneumothorax	ALS
Paul 2013 [17]	>28 d	<32 wGA Born in hospital RDS No weight limit BPD: O_2_ > 21% for ≥28 d + O_2_ < 30% at 36 wk PCA (moderate) or +O_2_ ≥ 30% and/or ventilation/CPAP at 36 weeks PCA ALS: pneumothorax or PIE	Total = 415 B 100/100 mg/kg = 201 P 200/100 mg/kg = 214	Mean wGA, 27.2 Mean weight, 1037 g Cesarean, 56.1% Male, 51.6% Prenatal steroids, 95.9% FiO_2_, no data Mean surfactant doses, B = 1.78, P = 1.63	B = 15/201 (7%) P = 24/214 (11%) Adjusted OR (CI)^a^, 0.63 (0.27–1.39) *P* = 0.239	B = 59/200 (29%) P = 78/212 (37%) Adjusted OR (CI)^a^, 0.73 (0.41–1.30) *P* = 0.282	B = 46/200 (23%) P = 63/212 (30%) Adjusted OR (CI)^a^, 0.82 (0.48–1.39) *P* = 0.455	No data	B = 11/201 (5.4%) P = 5/214 (2.3%) Adjusted OR (CI)^a^, 3.13 (0.95–11.11) *P* = 0.060
Ramanathan 2013 [16]	Until discharge	25–32 wGA Born in hospital RDS Weight 500–1999 g Age ≤ 2 d at first dose No congenital abnormalities	Total = 14,173 B = 5698 P = 5097 C = 3378	Mean wGA, no data Mean weight, no data Cesarean, no data Male, 54.6% Prenatal steroids, no data FiO_2_, no data Surfactant dosing, no data	B = 261/5698 (4.58%) P = 184/5097 (3.61%) C = 201/3378 (5.95%) Adjusted OR (95% CI)^b^: B vs. P, 1.370 (0.996–1.885), *P* = 0.053 C vs. P, 1.496 (1.014–2.209), *P* = 0.043 C vs. B, 1.092 (0.765–1.559), *P* = 0.626	No data	No data	No data	No data
Trembath 2013 [15]	>34 d	<37 wGA NICUs with ≥ 50 pts No multiple surfactants No weight limit No explicit requirement for RDS ALS: pneumothorax or PIE after first exposure to surfactant BPD: if <32 wGA, continuous O_2_ or respiratory support from 36–<37 wGA; if ≥32 wGA, from 28–34 days after birth	Total = 51,282 B = 20 383 P = 15 151 C = 15 748	Median wGA, 30 Median weight, 1435 g Cesarean, 72.8% Male, 57.0% Prenatal steroids, 63.7% FiO_2_, no data Surfactant dosing, no data	B = 2052 (10.1%) P = 1086 (7.2%) C = 1438 (9.1%) Adjusted OR (95% CI)^c^: B vs. P, 0.86 (0.72–1.04) C vs. B, 1.14 (0.93–1.39) C vs. P, 0.98 (0.78–1.23)	B = 5403 (27.4%) P = 2913 (19.9%) C = 3848 (24.9%) Adjusted OR (95% CI)^c^: B vs. P, 1.10 (0.96–1.27) C vs. B, 1.08 (0.93–1.26) C vs. P, 1.19 (1.00–1.41)	B = 3475 (17.6%) P = 1889 (12.9%) C = 2480 (16.1%) Unadjusted OR (95% CI), not analyzed	B = 1230 (6.0%) P = 616 (4.1%) C = 775 (4.9%) Unadjusted OR (95% CI), not analyzed	B = 1589 (7.8%) P = 802 (5.3%) C = 1059 (6.7%) Adjusted OR (95% CI)^c^: B vs. P, 1.06 (0.87–1.29) C vs. B, 1.17 (0.95–1.43) C vs. P, 1.23 (0.98–1.56)
Naseh 2014 [18]	Until discharge	<37 wGA RDS No weight limit No congenital heart disease or chromosomal anomalies	Total = 242 B 100 mg/kg = 74 P 100 mg/kg = 168	Mean wGA, 32.7 wk Mean weight, 2027 g Cesarean, 82.6% Male, 64.0% Prenatal steroids, 91.7% FiO_2_, no data Surfactant dosing, no data	B = 2/74 (2.7%) P = 19/168 (11.3%) *P* = 0.027	No data	No data	No data	No data

*ALS* air leak syndrome, *B* beractant, *BPD* bronchopulmonary dysplasia (also called chronic lung disease), *C* calfactant, *CI* confidence interval, *CPAP* continuous positive airway pressure, FiO_2_ fraction of inspired oxygen, *NICU* neonatal intensive care unit, *OR* odds ratio, P poractant alfa, *PCA* postconceptional age, *PIE* pulmonary interstitial emphysema, *pt* patient, *RDS* respiratory distress syndrome, *wGA* weeks of gestational age.

^a^The factors used for adjustment were gestational age, birthweight, antenatal steroids, chorioamnionitis, cesarean section, Apgar scores at 5 min, admission temperature, gender, cord pH, worst base deficit in the first hour of life, time of administration of surfactant, air leak, pulmonary hemorrhage, and patent ductus arteriosus requiring treatment.

^b^The factors used for adjustment were gestational age (categorized into 2-week groups, from 25–26 to 31–32 weeks), body weight (categorized into 250-g groups, from 500–749 to 1750–1999 g), gender, race, 3M All Patient Refined Diagnosis Related Group severity of illness category and risk of mortality category, US Census region, population served (urban/rural), teaching status (teaching/non-teaching), and hospital size (categorization based on the number of beds).

^c^The factors used for adjustment were gestational age, antenatal steroids, small-for-gestational-age status, and discharge year (other factors were tried but discarded).

#### Patient outcomes

**Fig. 2:**
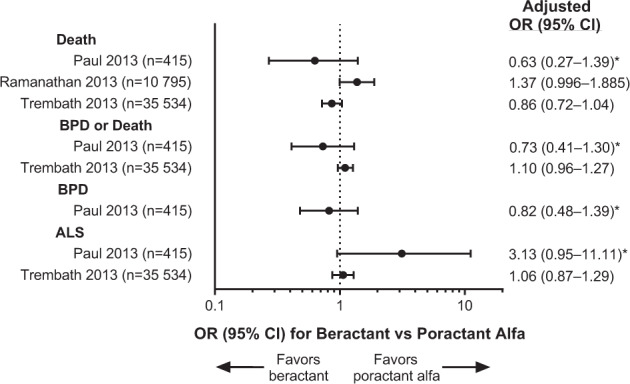
Forest plot of patient outcomes from RWE studies that reported adjusted analyses. The numbers of patients who received beractant or poractant alfa (but not other surfactants) are shown. The ORs and 95% CIs are given based on the values from the original articles, which had been adjusted for patient characteristics and other factors. Note: only 1 study specifically compared a 100-mg/kg dose for each surfactant [18]. ALS air leak syndrome, BPD bronchopulmonary dysplasia, CI confidence interval, OR odds ratio, RWE real-world evidence. *The factors used for adjustment were gestational age, birthweight, antenatal steroids, chorioamnionitis, cesarean section, Apgar scores at 5 min, admission temperature, gender, cord pH, worst base deficit in the first hour of life, time of administration of surfactant, air leak, pulmonary hemorrhage, and patent ductus arteriosus requiring treatment. ^†^The factors used for adjustment were gestational age (categorized into 2-week groups, from 25–26 to 31–32 weeks), body weight (categorized into 250-g groups, from 500–749 to 1750–1999 g), gender, race, 3M All Patient Refined Diagnosis Related Group severity of illness category and risk of mortality category, US Census region, population served (urban/rural), teaching status (teaching/non-teaching), and hospital size (categorization based on the number of beds). ^‡^The factors used for adjustment were gestational age, antenatal steroids, small-for-gestational-age status, and discharge year (other factors were tried but discarded).

Deaths were reported in all four RWE studies (Table 1; Fig. 2). The incidence of death was similar between the beractant and poractant alfa groups in three studies [15–17] but significantly lower with beractant versus poractant alfa in 1 study (2/74 [2.7%] vs. 19/168 [11.3%]; *P* = 0.027), which was also the only study that specifically compared a 100-mg/kg dose for each surfactant [18]; a 100 mg/kg dose of poractant alfa is not the currently approved dosage [6]. The incidence of BPD was reported in two studies [15, 17] and was similar in the beractant and poractant alfa groups in the 1 study that tested for a significant difference between treatments for this outcome [17]. The composite endpoint of BPD or death occurred with similar incidence in the beractant and poractant alfa groups in the two studies that reported this outcome [15, 17]. Pneumothorax was reported in 1 study, but there was no statistical test for that outcome [15]. The incidence of ALS was reported in two studies and was similar in the beractant and poractant alfa groups in both reports [15, 17].

### Randomized controlled trials

#### Study and patient characteristics

Fifteen RCTs were analyzed (Table 2) [19–33]. The follow-up times for study outcomes were generally not explicitly defined but appeared to range from 72 h to ≥28 days or >36 weeks PCA for some endpoints. Fourteen studies had mid-term to long-term outcomes (>72 h) [19, 20, 22–33], and 1 study had short-term outcomes (≤72 h) [21]. The criterion for gestational age was <37 or ≤37 wGA in five studies [22, 24, 25, 29, 32], <35 wGA in two studies [19, 26], ≤32 wGA in three studies [20, 27, 28], <30 wGA in one study [31], 26–36 wGA in one study [33], and 24 to <30 wGA in one study [21]. Two studies simply stated that patients had to be preterm, without defining a specific gestational age [23, 30]. Four studies imposed a limit on birth weight for enrollment: 700–1500 g [30], 750–1750 g [19], >750 g [26], and ≤2000 g [28]. The studies included between 30 and 293 patients. Nine studies included data about doses that were administered according to the US product label (i.e., beractant 100 mg/kg; poractant alfa 200 mg/kg for the first dose, and 100 mg/kg for subsequent doses) [19, 20, 22, 23, 25, 27, 29, 31, 33]. Six studies specifically compared beractant 100 mg/kg with poractant alfa 100 mg/kg [19, 24, 26, 28, 30, 32]. Median or mean gestational age ranged from ~26 to 33 weeks. Median or mean birth weight ranged widely among studies, from 731 to 1911 g, trending higher with greater gestational age. The proportions of patients who were delivered by Cesarean section ranged from 57.6% to 83.0%. The proportion of male patients ranged from 41.3% to 66.7%. The use of prenatal steroids ranged from low to nearly universal (range, 30.0–98.1%). Baseline values for FiO_2_ in the five studies for which data were reported ranged widely, from ~0.5 to 0.9 [19, 23, 28–30]. Six studies reported the mean number of doses of surfactant (range, 1.06–2.2) [22, 23, 25, 26, 29, 30] and six studies reported the proportion of patients who received >1 dose (range, 13–68%) [19, 21, 24, 27, 28, 31]; by either measure, more doses of beractant than poractant alfa were given in most studies. Three studies had BPD data at 28 weeks after birth [19, 22, 32], and six studies assessed BPD at 36 weeks PCA or PMA [23, 27–31].

**Table 2:** Randomized controlled trials.

Study and blinding	Follow-up	Key criteria	Population	Characteristics	Outcomes			
					Death	BPD	Pneumothorax	ALS
Speer 1995 [30] Some assessments blinded	Long-term, 28 d	Preterm Weight 700–1500 g RDS Age 1–24 h BPD: O_2_ at 36 wk PCA	Total = 73 B 100 mg/kg = 40^a^ P 100 mg/kg = 33^a^	Mean wGA, 28.8 Mean weight, 1088.2 g Cesarean, no data Male, 45.2% Prenatal steroids, 49.3% Median FiO_2_, B = 0.9, P = 0.9 Mean doses, B = 2.2, P = 1.7	B = 5/40^a,b^ P = 1/33^a,b^	B = 4/40^a,b,c^ P = 4/33^a,b,c^	B = 5/40^a,b^ P = 2/33^a,b^	PIE B = 4/40^a,b^ P = 1/33^a,b^
Baroutis 2003 [28] Blinded for A and P but not B	Long-term, >36 weeks PCA or discharge	≤32 wGA Weight ≤ 2000 g Born in center With RDS No major congenital anomalies ALS: PIE or pneumothorax BPD: O_2_ at > 36 weeks PCA	Total = 80 B 100 mg/kg = 26^a^ P 100 mg/kg = 27^a^ A 100 mg/kg = 27^d^	Mean wGA, 29.0 Mean weight, 1203 g Cesarean, 61.3% Male, 51.3% Prenatal steroids, 30.0% Mean FiO_2_, 0.64 s dose, B = 23.1%, P = 14.8%, A = 18.5%	B = 6/26^a,b^ P = 5/27^a,b^ A = 7/27^d^	B = 4/26^a,b,c^ P = 4/27^a,b,c^ A = 3/27^d^	No data	B = 4/26^a,b^ P = 3/27^a,b^ A = 2/27^d^
Ramanathan 2004 [19] Observer blinded on day 1, otherwise OL	Long-term, 28 d (all patients) or until 36 wk PCA (if born at ≤32 wGA)	<35 wGA Birth weight 750–1750 g Evidence of RDS Intubated and mechanically ventilated Age < 6 h at randomization FiO_2_ ≥ 0.30 and O_2_ saturation 88%–95% No life-threatening congenital anomalies	Total = 293 (<35 wGA) Initial/later dose B 100/100 mg/kg = 98^a,b^ P 100/100 mg/kg = 96^a^ P 200/100 mg/kg = 99^b^ Total = 270 (≤32 wGA)^d^ B 100/100 mg/kg = 90^d^ P 100/100 mg/kg = 85^d^ P 200/100 mg/kg = 95^d^	Mean wGA, 28.7 Mean weight, 1162 g Cesarean, no data Male, 59.0% Prenatal steroids, 80.9% Mean FiO_2_, approximately 0.63 > 1 dose, B = 68%, P = 36%	At day 28 B = 8/98^a,b,e^ P 100 = 6/96^a,c^ P 200 = 3/99^a,e^ At 36 weeks PCA^d^ B = 10/90^d^ P 100 = 9/85^d^ P 200 = 3/95^d^	At day 28 B = 49/98^a,b,e,f^ P 100 = 48/96^a,b,f^ P 200 = 49/99^b,e,f^ At 36 weeks PCA^d^ B = 39/90^d^ P 100 = 40/85^d^ P 200 = 37/95^d^	At day 28 B = 5/98^a,e^ P 100 = 6/96^a,b^ P 200 = 3/99^b,e^ At 36 weeks PCA^d^ B = 5/90^d^ P 100 = 5/85^d^ P 200 = 3/95^d^	No data
Malloy 2005 [29] Some assessments blinded	Long-term, ≤40 corrected wGA	<37 wGA With RDS No weight limit BPD: O_2_ at 36 wk PMA and ≥28 d of age	Total = 58 B 100 mg/kg = 29^b^ P 200 mg/kg = 29^b^	Mean wGA, 29.5 Mean weight, 1401 g Cesarean, no data Male, 46.6% Prenatal steroids, 74.1% Mean FiO_2_, 0.48 Mean doses, B = 1.7, P = 1.2	B = 3/29^b,e^ P = 0/29^b,e^	B = 10/27^b,e,f^ P = 10/29^b,e,f^	B = 1/29^b,e^ P = 2/29^b,e^	No data
Gharehbaghi 2010 [23] Blind	Long-term, 7 d or until discharge	Preterm with RDS No major congenital anomalies No weight limit ALS: PIE or pneumothorax BPD: O_2_ at > 36 wk PCA	Total = 150 B 100 mg/kg = 71^b^ P 200 mg/kg = 79^b^	Mean wGA, 29.45 Mean weight, 1444.4 g Cesarean, 65.3% Male, 58.7% Prenatal steroids, 44.0% Mean FiO_2_, 0.72 Mean surfactant doses, B = 1.06, P = 1.06	B = 15/71^b,e^ P = 21/79^b,e^	B = 20/71^b,c,e^ P = 20/79^b,c,e^	No data	B = 5/71^b,e^ P = 2/79^b,e^
Mercado 2010 [31] OL	Long-term, follow-up: ≥36 wk PMA (BPD)	wGA < 30 RDS No multiple congenital abnormalities No weight limit BPD: O_2_ at 36 wk PMA	Total = 40 B 100/100 mg/kg = 20^b^ P 200/100 mg/kg = 20^b^	Mean wGA, 26 Mean weight, 731 g Cesarean, no data Male, 45.0% Prenatal steroids, 95.0% Mean FiO_2_, no data > 1 dose, B = 45.0%, P = 45.0%	B = 1/20^b,e^ P = 4/20^b,e^	B = 13/20^b,c,e^ P = 8/20^b,c,e^	No data	No data
Dizdar 2012 [25] Blinding not specified	Long-term, 40 wk corrected gestational age	wGA < 37 RDS within 6 h of birth No weight limit No congenital heart or lung diseases No weight limit BPD per US NIH criteria	Total = 126 B 100/100 mg/kg = 65^b^ P 200/100 mg/kg = 61^b^	Median wGA, 28 Median weight, 1080–1165 g Cesarean, 81.7% Male, 57.9% Prenatal steroids, 56.3% FiO_2_, no data Mean doses, B = 1.34, P = 1.11	B = 13/65^b,e^ P = 6/61^b,e^	B = 18/65^b,e^ P = 9/61^b,e^	B = 3/65^b,e^ P = 4/61^b,e^	No data
Saeidi 2013 [32] Blinding not specified	Long-term, ≥28 d (BPD)	<37 wGA RDS No weight limit No major congenital anomalies Age ≤ 4 h BPD: O_2_ at 28 d	Total = 104 B 100/100 mg/kg = 74^a^ P 100/100 mg/kg = 30^a^	Mean wGA, 29.84 Mean weight, 1360 g Cesarean, 63.5% Male, 41.3% Prenatal steroids, no data FiO_2_, no data Repeat surfactant dosing, no data	B = 21/74^a,b^ P = 8/30^a,b^	B = 30/74^a,b,f^ P = 12/30^a,b,f^	B = 15/74^a,b^ P = 6/30^a,b^	No data
Eras 2014 [20] OL	Long-term, until 18–24 mo corrected age	≤32 wGA RDS No weight limit No major congenital anomalies	Total = 215 B = 102^b^ P = 113^b^	Mean wGA, 28.5 Mean weight, 1128 g Cesarean, no data Male, 48.4% Prenatal steroids, no data FiO_2_, no data Repeat surfactant dosing, no data	B = 10/125^b,e^ P = 7/135^b,e^	B = 28/102^b,e^ P = 24/113^b,e^	No data	No data
Karadag 2014 [27] Blinding not specified	Long-term, 36 wk PMA	wGA ≤ 32 Born in hospital RDS Age ≤ 2 h No major congenital anomalies No weight limit BPD: O_2_ at 36 wk PMA	Total = 92 B 100/100 mg/kg = 46^a^ P 200/100 mg/kg = 46^a^	Mean wGA, 29.3 Mean weight, 1092 g Cesarean, 57.6% Male, 55.4% Prenatal steroids, 79.3% FiO_2_, no data Second dose, B = 47.8%, P = 19.6%	B = 8/46^b,e^ P = 4/46^b,e^	B = 9/46^b,c,e^ P = 6/46^b,c,e^	B = 6/46^b,e^ P = 2/46^b,e^	No data
Terek 2015 [33] OL	Long-term, 6 h for oxygenation and hemodynamics assessments, otherwise not stated	26–36 wGA RDS Born in hospital No weight limit No congenital heart or lung diseases No major congenital anomalies	Total = 30 B 100 mg/kg = 15^b^ P 200 mg/kg = 15^b^	Mean wGA, 29.8 Mean weight, 1396.7 g Cesarean, no data Male, 46.7% Prenatal steroids, 72% Mean FiO2, 0.6887 Repeat surfactant dosing, no data	B = 3/15^b,e^ P = 4/15^b,e^	B = 4/15^b,e^ P = 4/15^b,e^	B = 0/15^b,e^ P = 0/15^b,e^	No data
Mussavi 2016 [22] Triple-blind	Long-term, ≥28 d (BPD)	≤37 wGA Admitted to NICU with RDS No weight limit Age ≤ 6 h No congenital anomalies BPD: O_2_ at ≥ 28 d Pneumothorax: air leak that accumulated in pleural space	Total = 165 B 100 mg/kg = 62^b^ P 200 mg/kg = 54^b^ A 100 mg/kg = 49^d^	Mean wGA, 31.6 Mean weight, 1840 g Cesarean, 69.1% Male, 66.7% Prenatal steroids, no data FiO_2_, no data Mean surfactant doses, B = 1.08, P = 1.06, A = 1.55	No data on post-intervention deaths	B = 2/62^b,e,f^ P = 1/54^b,e,f^ A = 1/49^f^	B = 2/62^b,e^ P=3/54^b,e^ A=7/49^f^	No data
Najafian 2016 [26] Blinding not specified	Long-term, until discharge	wGA < 35 Born in hospital RDS Age ≤ 6 h No congenital heart diseases or life-threatening congenital anomalies Weight >750 g	Total = 112 B 100 mg/kg=56^a^ P 100 mg/kg=56^a^	Mean wGA, 32.59 Mean weight, 1911.3 g Cesarean, 83.0% Male, 58.0% Prenatal steroids, 72.3% FiO_2_, no data Mean doses, B = 1.32, P = 1.18	B = 6/56^a,b^ P = 2/56^a,b^	No data	B = 5/56^a,b^ P = 2/56^a,b^	No data
Mirzarahimi 2018 [24] Blind	Long-term, ≥28 d (BPD)	wGA < 37 with RDS No weight limit No congenital anomalies BPD: O_2_ at 28 d	Total = 150 B 100 mg/kg = 75^a^ P 100 mg/kg = 75^a^	Mean wGA, 29.65 Mean weight, no data Cesarean, no data Male, 46.7% Prenatal steroids, no data FiO_2_, no data >1 surfactant dose, B = 28.0%, P = 13.3%	B = 15/75^a,b^ P = 13/75^a,b^	12% (no comparison)^d,f^	16.7% (no comparison)^d^	No data
Fujii 2010 [21] OL	Short-term, 72 h after birth for level of respiratory support during NICU hospitalization for morbidities of prematurity	24–<30 wGA Born in hospital RDS requiring ventilation No weight limit Age < 6 h at randomization No severe congenital anomalies, significant CHD, or death expected in ≤3 d ALS: not defined BPD: O_2_ at 36 wk PCA Observation time for BPD: 72 h	Total = 52 Initial/later dose B 100/100 mg/kg = 27^d^ P 200/100 mg/kg = 25^d^	Mean wGA, 26.9 Mean weight, 914 g Cesarean, 69.2% Male, 61.5% Prenatal steroids, 98.1% FiO_2_, no data >1 dose, B = 56%, P = 36%	B = 5/27 (19%)^d^ P = 2/25 (8%)^d^	B = 11/22 (50%)^d^ P = 8/23 (35%)^d^	No data	B = 4/27 (15%)^d^ P = 0/25 (0%)^d^

*A* Alveofact (bovactant), *ALS* air leak syndrome, *B* beractant, *BPD* bronchopulmonary dysplasia (also called chronic lung disease), *CHD* congenital heart disease, *FiO*_2_ fraction of inspired oxygen, *NICU* neonatal intensive care unit, *NIH* National Institutes of Health, *OL* open label, *P* poractant alfa, *PCA* postconceptional age, *PIE* pulmonary interstitial emphysema, *PMA* postmenstrual age, *RDS* respiratory distress syndrome, *wGA* weeks of gestational age.

^a^Data on B 100 mg/kg vs. P 100 mg/kg (i.e., excluding P 200 mg/kg) were used for additional sensitivity analyses.

^b^Data were used for primary meta-analyses.

^c^Data for which BPD was assessed at 36 weeks PCA or PMA were used for additional sensitivity analyses.

^d^Data not included in any current analyses.

^e^Data for on-label doses were used for sensitivity analyses.

^f^Data for which BPD was defined as the need for oxygen at 28 days after birth were used for additional sensitivity analyses.

Eight RCTs were included in the previous meta-analysis conducted by Singh et al.; we confirmed the grading conferred by the authors of that publication for those eight studies. Grading for the additional seven studies identified in the current meta-analysis is presented in Supplementary Table 2.

#### Patient outcomes

Thirteen mid-term to long-term RCTs of beractant and poractant alfa provided data on death [19, 20, 23–33], 12 on BPD [19, 20, 22, 23, 25, 27–33], 9 on pneumothorax [19, 22, 25–27, 29, 30, 32, 33], and 3 on ALS [23, 28, 30]; the short-term RCT, not included in the meta-analyses, provided data on death, BPD, and ALS (Table 2) [21].

**Fig. 3:**
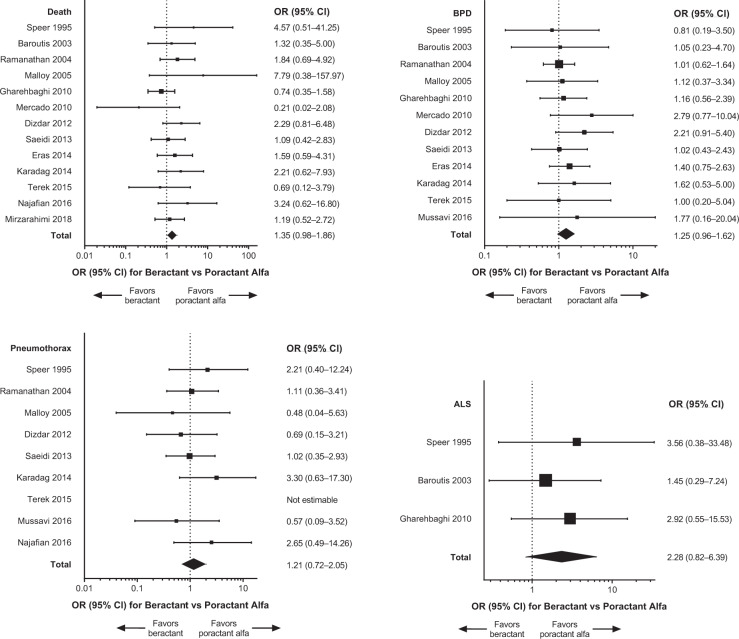
Forest plots of patient outcomes from primary meta-analyses of RCTs, with any dose of surfactant. ALS air leak syndrome, BPD bronchopulmonary dysplasia, CI confidence interval, OR odds ratio, RCT randomized controlled trial.

**Fig. 4:**
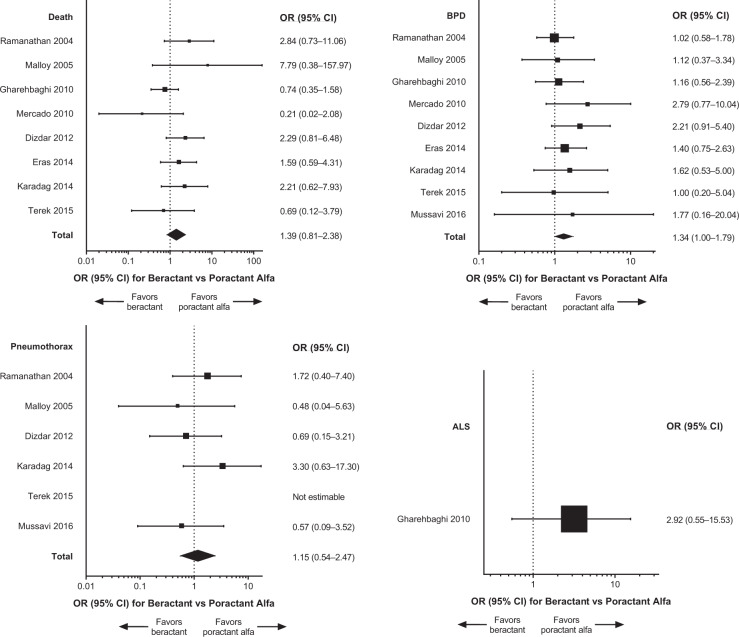
Forest plots of patient outcomes from sensitivity analysis that included only data from RCTs or RCT treatment arms in which doses of surfactant were given according to the US product label. ALS air leak syndrome, BPD bronchopulmonary dysplasia, CI confidence interval, OR odds ratio, RCT randomized controlled trial.

**Fig. 5:**
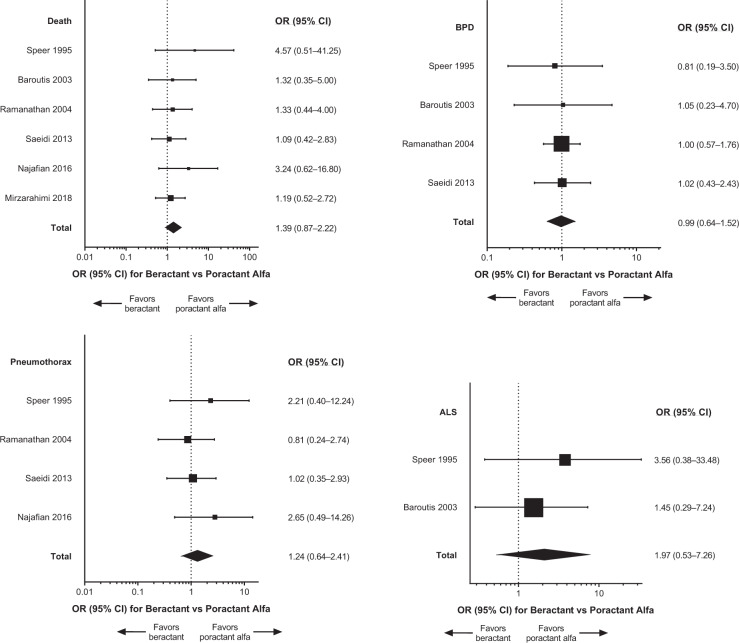
Forest plots of patient outcomes from sensitivity analysis that included only data from RCTs or RCT treatment arms with 100 mg/kg doses of surfactant. ALS air leak syndrome, BPD bronchopulmonary dysplasia, CI confidence interval, OR odds ratio, RCT randomized controlled trial.

**Fig. 6:**
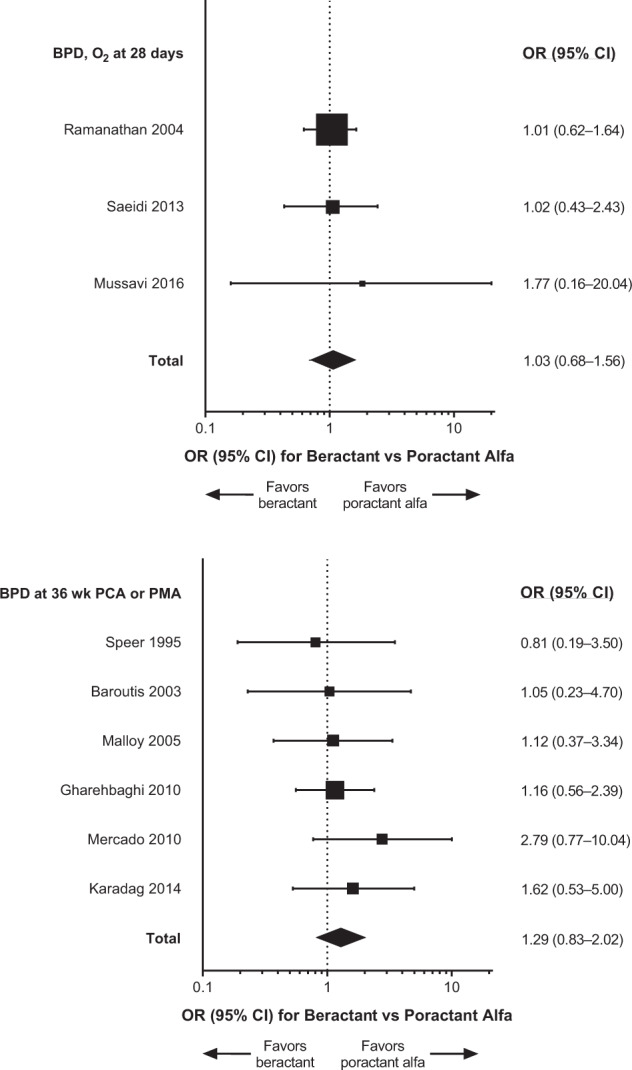
Forest plots BPD from sensitivity analyses that included only data from RCTs in which BPD defined as the need for oxygen at day 28 after birth or evaluated at 36 weeks PCA or PMA. BPD bronchopulmonary dysplasia, CI confidence interval, OR odds ratio, PCA postconceptional age, PMA postmenstrual age, RCT randomized controlled trial.

Primary meta-analysis results and individual studies demonstrated no significant differences (i.e., 95% CI of the OR encompassed 1) between treatment with beractant compared with poractant alfa for death, BPD, pneumothorax, and ALS (Fig. 3). The sensitivity analysis that included only data from doses of surfactant that complied with the US product labels (*n* = 9 studies) generally supported the findings of the primary meta-analyses [19, 20, 22, 23, 25, 27, 29, 31, 33]; the comparisons between beractant and poractant alfa were not statistically significant for death, pneumothorax, and ALS (Fig. 4). The incidence of BPD was of borderline significance (overall treatment effect, *P* = 0.05) overall but not in individual studies (Fig. 4). The risk difference of BPD estimated in the sensitivity analysis was 0.04 (95% CI: −0.00 to 0.08; *P* = 0.07). The sensitivity analysis that compared only data about beractant 100 mg/kg versus poractant alfa 100 mg/kg (*n* = 6 studies [19, 24, 26, 28, 30, 32]) revealed no significant differences in outcomes in any individual study or the meta-analyses (Fig. 5). In the sensitivity analyses of data for which BPD was defined as the need for oxygen at day 28 after birth (*n* = 3 studies [19, 22, 32]) or evaluated at 36 weeks PCA or PMA (*n* = 6 studies [23, 27–31]), the overall analyses and individual study results did not demonstrate significant differences between beractant and poractant alfa (all doses of beractant and poractant alfa included; Fig. 6).

The original publication of the short-term (<72 h of follow up) RCT, which was not included in the meta-analyses, reported no significant differences between treatments for the incidences of death and BPD, whereas there was a trend favoring poractant alfa over beractant for prevention of ALS [21].

## Discussion

The objective of this analysis was to compare clinical outcomes with beractant and poractant alfa using the most current published data from RWE studies and RCTs in premature infants treated for acute RDS. The primary results indicated that outcomes were not statistically significantly different in almost all individual RWE studies and RCTs, and that death, BPD, pneumothorax, and ALS occurred with similar incidence in the meta-analyses of RCTs. The only RWE study that compared the incidence of BPD with beractant and poractant alfa found no significant difference between treatments [17].

RWE studies and RCTs provide complementary information; RWE studies are more representative of real-world clinical practice, whereas RCTs are more rigorously controlled for factors other than the treatment of interest. A full understanding of the relative utility of a therapy is best derived from both kinds of evidence. However, comparison of the results of the RWE studies and the RCTs is complicated by several factors. There was substantial variation among the analyzed studies in design, entry criteria, demographic and disease characteristics, dosing regimens, definitions and reporting of endpoints, and follow-up time. The RWE studies were reported during a narrow range of time (2013–2014), whereas the RCTs were reported over a much longer period (1995–2018); thus, it is possible that aspects of therapy, including evolution of care, other than the type of surfactant may have differed. Treatment in the RWE studies was not randomized and was always open-label, whereas it was sometimes blinded in the RCTs. Finally, the populations of two of the RWE studies were far larger than the populations of any of the RCTs, resulting in much greater power to detect statistical differences between groups; counteracting this was the greater heterogeneity of the RWE populations due to lack of restrictions in inclusion criteria, controlled selection of the population enrolled, and treatment approach, although this was at least partially compensated via adjusted analyses in the original articles. Despite these caveats, the general consistency of the results among RWE studies and the meta-analyses of RCTs suggest that the findings were valid. Further large-scale research could shed more light on whether there is a real-world difference between beractant and poractant alfa treatment for the outcome of BPD. The monitoring of post-marketing safety would provide relevant information to regulatory agencies and would be essential in updating US product labels, as needed. Given the vulnerable population of premature neonates and the criticality of RDS, as well as the evolution of standard of care in the last 20 years, it is relevant and critical to continue to monitor and update healthcare professionals on the safety of these products, particularly in real-world scenarios.

Our RCT meta-analysis results were in agreement with the findings of a previous meta-analysis reported by Singh et al. [9] for several outcomes but differed for the risk of death. We found no significant difference between beractant and poractant alfa in the risk of death, whereas Singh et al. reported an increased risk of death with beractant compared with poractant alfa (risk ratio [95% CI], 1.44 [1.04–2.00]) [9]. However, the difference in mortality reported by Singh et al. was observed only for higher doses of poractant alfa, whereas our own findings were consistent regardless of dosing regimen. Additionally, the network meta-analysis of Zhang et al., which included only death as an outcome, agreed with our results, finding no significant difference in mortality between treatment with beractant and poractant alfa (OR [95% CI], 1.27 [0.99–1.62]) [10]. Our primary finding for BPD and sensitivity analyses based on specific definitions were comparable to the conclusion of Singh et al. that there was a similar risk of BPD with either treatment according to two different definitions of BPD (need for oxygen at 28–30 days old, risk ratio [95% CI], 0.97 [0.77–1.23]; need for oxygen at 36 weeks PMA, 0.94 [0.79–1.12]). Our results agree with those of Singh et al. for the risks of pneumothorax and ALS in that there was no significant difference between beractant and poractant alfa. A recent meta-analysis published by Tridente et al. in 2019 [34] concluded that poractant alfa was significantly more effective than bovine-derived surfactants at prevention of BPD in preterm infants with RDS, but that finding is not comparable to our work because it was based on combined data from beractant and two other bovine-derived surfactants (bovactant and bovine lipid extract surfactant suspension) that may have nonequivalent properties.

Among the main factors that could have led to differences in conclusions among meta-analyses are the number and types of studies that were pooled. We included some of the same studies as the previous meta-analyses of Singh et al. and Zhang et al. [9, 10]. However, we also added 9 studies that were not represented in either of the earlier meta-analyses; these included 2 RWE studies [17, 18] and 7 RCTs [20, 22, 24, 26, 31–33]. The seven additional RCTs contributed a total of 816 patients to the pooled meta-analyses of treatment with beractant or poractant alfa, comprised over half of the 13 RCTs that we included for meta-analysis of the risk of death, and were on average more recent (published in 2010–2018) compared with the studies analyzed by Singh et al. (1995–2014) or Zhang et al. (1995–2013) [9]. Six studies were included in all three works (and in meta-analyses in each case, except for data from Fujii et al. in our work) [19, 21, 23, 25, 28, 30], two were included in the present article and by Singh et al. (and in meta-analyses) [27, 29], and two were included in the present article (not subjected to meta-analysis by us because they were RWE studies) and by Zhang et al. [15, 16]. Singh et al. included one article, a doctoral dissertation about a small study [35], which we did not because it was not published in a peer-reviewed format. Thus, the meta-analysis results reported here are founded upon a sample that was more robust and also likelier to reflect current clinical practice compared with the previous meta-analysis publications. A final aspect that might have influenced the findings in our meta-analyses as compared with prior publications was the choice, when similar endpoints were reported (e.g., death at 28 days after birth or at 36 weeks PCA, as in Ramanathan et al. 2004 [19]), of which one to subject to meta-analysis. We chose the former outcome (death at 28 days) because it appeared more applicable across the studies available for pooling.

Despite the increased power to observe treatment differences that is afforded by comparing studies or pooling multiple trials in a meta-analysis, any such efforts have limitations. For the RWE studies, the limitation of study heterogeneity was substantial enough that meta-analysis was not attempted. For example, in the studies analyzed here, the follow-up period was often vague or differed between studies. Normally, studies follow up only as long as patients are in the neonatal intensive care unit (NICU), where patients typically reside for about 1 month. BPD, pneumothorax, and ALS are usually observed within a 1-month timeframe. Any deaths during the period are noted. Patients who survive their first month typically leave the NICU. The studies also had variable inclusion criteria that were reflected in the characteristics of the study populations; for example, there were large differences in gestational age cutoffs and, as a result, birth weights. The definitions of outcomes were frequently not uniform; however, this reflects the reality that clinical practices are themselves diverse. Some outcomes (e.g., ALS) were sporadically reported, reducing the power to detect differences between treatment groups. Doses of surfactant did not always match the US product label recommendations, although the similar findings among the meta-analyses that included all doses and the sensitivity analyses that included only doses that matched the US product label argue against a prominent effect, and off-label dosing also reflects clinical practice. Also, with regards to death, BPD, and pneumothorax, the ORs and corresponding 95% CIs for beractant 100 mg/kg vs. poractant alfa 100 mg/kg in our analyses were 1.39 (0.87–2.22), 0.99 (0.64–1.52), and 1.24 (0.64–2.41), respectively, emphasizing the similar efficacy of both products. However, the studies comparing beractant 100 mg/kg with poractant alfa 100 mg/kg, although they did not demonstrate significant differences between treatments in these sensitivity analyses, were largely limited to research that was older or conducted under circumstances in which the dose regimen may have been chosen due to limited resources. Current practice is dominated by administration of poractant alfa at its recommended initial dose (200 mg/kg; 2.5 mL/kg) rather than at a lower dose (100 mg/kg; 1.25 mL/kg), and so with a volume by patient weight that is similar to that for beractant 100 mg/kg (4 mL/kg). Although a slightly larger volume is used with beractant, the amount of total phospholipid received is consistent with the normal content of phospholipid in the natural surfactant produced by at-term infants [36]. When 100 mg of phospholipid is used with poractant alfa, even though the amount of phospholipid is similar to physiologic conditions, the clinical outcome does not appear to be similar to beractant, at least with regards to the incidence of death. This might be due to a smaller volume of distribution (1.25 vs. 4 mL). Although there is a larger exposure to phospholipids than in physiologic conditions, when the volume of poractant alfa is increased to 2.5 mg/kg (i.e., 200 mg/kg), there is an evident improvement in clinical outcomes (in particular with incidence of death), and is thus comparable to beractant. All sensitivity analyses were limited by smaller sample sizes relative to the primary analyses, with the result that an absence of significance could have reflected the lesser statistical power. A final limitation is that, for RCTs of surfactants, full blinding is generally impossible because of different volumes and methods of administration among products, which might have resulted in bias among study personnel. Regardless of these potential limitations, the consistency of the findings supports the overall validity of the findings. Methods of less invasive surfactant administration might improve some outcomes as they become more widely adopted [37–39].

The systematic review of RWE studies and our primary meta-analysis of RCTs indicate that the incidences of mortality, BPD, pneumothorax [40], and ALS for beractant compared with poractant alfa were similar in premature infants treated for acute RDS. The results of our primary meta-analysis of RCTs was in agreement with that reported by Zhang et al. [10], which only included death as an outcome, and with Singh et al. [9], for all the aforementioned outcomes, except for risk of death. Although there are inherent limitations to RWE studies (namely study heterogeneity, greater heterogeneity of the RWE populations due to lack of randomization, and variation in the definitions of outcomes), RWE studies are more representative of and more accurately reflect the experiences of patients in usual clinical practice. In the RWE studies, no significant differences in outcomes were observed between beractant and poractant alfa, suggesting that there may be no meaningful real-world differences between these treatments.

## Supplementary information

Supplemental Material
