# Supplementary material for: Beractant and poractant alfa in premature neonates with respiratory distress syndrome: a systematic review of real-world evidence studies and randomized controlled trials
Source: J Perinatol. 2020 Feb 12;40(8):1121–34. doi: 10.1038/s41372-020-0603-7 (PMC7375954; doi:10.1038/s41372-020-0603-7)
Supplement: Supplementary file 1 — Supplemental Material [file 41372_2020_603_MOESM1_ESM.pdf]

Supplementary Information: This file is a Word document containing 2 tables with an assessment of study quality for studies included in this review.

Supplementary Table 1. Assessment of Study Quality for Real-World Evidence Studies

Study	Clear Objective	Randomized	Allocation Concealment	Blinded	Groups Similar at Baseline	Outcomes Reliable and Defined	Description of Withdrawals and Dropouts (If Applicable)	ITT Analysis
Naseh 2014	Yes	No; retrospective analysis	NA	NA	NS	Yes	NA	NA
Paul 2013	Yes	No; retrospective cohort analysis	NA	NA	Yes (except for gestational age)	Yes	NA	NA
Ramanathan 2013	Yes	No; retrospective cohort analysis	NA	NA	No	Yes	NA	NA
Trembath 2013	Yes	No; retrospective database analysis	NA	NA	No	Yes	NA	NA

	Considers Potential Confounders	Provides CIs
Naseh 2014	No	No
Paul 2013	Yes	Yes
Ramanathan 2013	Yes	Yes

Trembath 2013	Yes	Yes

CI, confidence interval; ITT, intent to treat; NA, not applicable; NS, not specified.

Clear objective: The study objective was clearly reported.

Randomized: The study was randomized.

Allocation concealment: The allocation concealment method was appropriate, such as the use of centralized or computerized allocation systems or the use of coded identical containers.

Blinded: The study was blinded.

Groups similar at baseline: Patient characteristics and demographics were similar between treatment groups at baseline before any treatment was administered.

Outcomes reliable and defined: Primary and secondary outcomes were clearly defined, reliably measured, and all reported.

Description of withdrawals and dropouts: Withdrawals and dropouts were clearly reported.

ITT analysis: Patients were analyzed in the groups to which they were randomly allocated.

Considers potential confounders: Potential confounders were identified and taken into account.

Provides CIs: Clarifies whether study reports 95% CIs for primary (and secondary) endpoints.

Supplementary Table 2. GRADE Evidence Profile for Randomized Controlled Trials

Study	Study Limitations	Imprecision	Inconsistency of Results	Indirectness of Evidence	Quality of Evidence (GRADE)
Mirzarahimi 2018	No serious limitations	No serious imprecision	No serious inconsistency	No serious indirectness	High
Mussavi 2016	No serious limitations	No serious imprecision	No serious inconsistency	No serious indirectness	High
Eras 2014	No serious limitations	No serious imprecision	No serious inconsistency	Serious indirectness (downgraded one level because BPD was assessed with no definition)	Moderate
Najafian 2016	Serious limitation (downgraded because it was not specified if the study was blinded)	No serious imprecision	No serious inconsistency	No serious indirectness	Moderate
Terek 2015	Serious limitation (downgraded because it was not a blinded study)	No serious imprecision	No serious inconsistency	No serious indirectness	Moderate
Mercado 2010	Serious limitation (downgraded because it was not a blinded study)	Serious imprecision (downgraded because no dropouts were reported and paper was not clear whether all enrolled patients were treated and analyzed)	No serious inconsistency	No serious indirectness	Low
Saeidi 2013	Serious limitation (downgraded because it was not specified if the study was blinded)	Serious imprecision (downgraded because paper reported limited details on baseline characteristics and/or dropouts)	No serious inconsistency	No serious indirectness	Low
